# An Outstanding Role of Adipose Tissue in Canine Stem Cell Therapy

**DOI:** 10.3390/ani12091088

**Published:** 2022-04-22

**Authors:** Marina Prišlin, Dunja Vlahović, Petar Kostešić, Ivana Ljolje, Dragan Brnić, Nenad Turk, Ivana Lojkić, Valentina Kunić, Tugomir Karadjole, Nina Krešić

**Affiliations:** 1 Croatian Veterinary Institute, Savska Cesta 143, 10000 Zagreb, Croatia; prislin@veinst.hr (M.P.); brnic@veinst.hr (D.B.); ilojkic@veinst.hr (I.L.); kunic@veinst.hr (V.K.); 2 Faculty of Veterinary Medicine, University of Zagreb, Heinzelova 55, 10000 Zagreb, Croatia; dvlahovic@vef.unizg.hr (D.V.); kostesic@vef.hr (P.K.); turk@vef.unizg.hr (N.T.); ktugomir@vef.unizg.hr (T.K.); 3 Veterinary Clinic for Small Animals Buba, Dore Pfanove 11, 10000 Zagreb, Croatia; ivana.ljolje@gmail.com

**Keywords:** canine, adipose-derived mesenchymal stem cells, stem cell therapy, regenerative veterinary medicine, stromal vascular fraction, adipose tissue

## Abstract

**Simple Summary:**

Throughout history, the role of adipose tissue has changed for humans, and regarding canines: the role has changed from connective tissue to restoration of physiological functions. The adipose tissue cells have extraordinary mechanisms of healing tissue function, and the most outstanding component of adipose tissue discovered are mesenchymal stem cells. It has been almost fifteen years since their discovery in canine adipose tissue. Since then, numerous studies have investigated the possibilities of adipose-derived mesenchymal stem cells in treating various canine diseases. This review summarised the progress of confirming the therapeutic role of adipose tissue components, focusing on stem cells as the most researched and with the highest potential in enabling a better quality of life for canines.

**Abstract:**

Adipose tissue, previously known as connective tissue with a role in energy storage, is currently changing the course of treatments in veterinary medicine. Recent studies have revealed one particularly impressive function among all the newly discovered functions of adipose tissue. The interactive cells hosted by adipose tissue, the stromal vascular fraction (SVF), and their role in treating numerous diseases have provided a prospective course of research with positive outcomes in regenerative veterinary medicine (RVM). This review describes the main features of adipose tissue, emphasizing an eclectic combination of cells within the SVF and its thus far researched therapeutic possibilities in canine RVM. An afterwards focus is on a highly researched component of the SVF, adipose-derived mesenchymal stem cells (ASCs), which were shown to have an extraordinary impact relying on several proposed mechanisms of action on mitigating pathologies in canines. Furthermore, ASC therapy showed the most significant results in the orthopaedics field and in neurology, dermatology, ophthalmology, gastroenterology, and hepatology, which elevates the possibilities of ASC therapy to a whole new level. Therefore, this review article aims to raise awareness of the importance of research on cellular components, within abundant and easily accessible adipose tissue, in the direction of regenerative therapy in canines, considering the positive outcomes so far. Although the focus is on the positive aspects of cellular therapy in canines, the researchers should not forget the importance of identifying the potential negative aspects within published and upcoming research. Safe and standardized treatment represents a fundamental prerequisite for positively impacting the lives of canine patients.

## 1. Introduction

Adipose tissue (AT) was once considered to be the only connective tissue involved in energy storage. Currently, recognition of AT function is beyond simple fat storage, and is well known as a metabolic and endocrine organ [1,2,3]. Consequently, this review aims to raise awareness on the importance of research on cellular components, within abundant and easily accessible adipose tissue, in the direction of regenerative therapy in canines, considering positive outcomes so far and failures of current treatment options.

**Figure 1:**
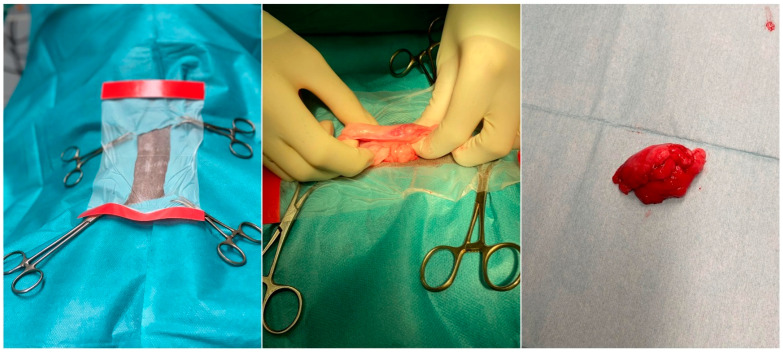
Adipose tissue collection during canine ovariotomy. The routine procedure commonly performed in young females presents an excellent opportunity to collect adipose tissue and store cells for future use in regenerative therapy.

Today it is accepted that adipose tissue is of mesodermal origin. However, there is evidence that craniofacial adipose deposits may originate from the neural crest [4]. Consequently, the origins of adipose tissue are complex and have to be fully explored [5]. Adipose tissue is crucial in maintaining lipid and glucose homeostasis [6]. Endocrine role turnover appears with the possibility of producing oestrogen, resistin, and leptin [1,7] and regulates food intake, body mass, reproductive functioning, foetal growth, pro-inflammatory immune responses, angiogenesis, and lipolysis [8]. Furthermore, it was discovered that AT secretes pro-inflammatory chemokines and cytokines such as interleukins (IL) 1, IL 6, IL 8, tumour necrosis factor-alpha (TNF-α), as well as proteins with a role in lipid metabolism, in vascular haemostasis or the complement system. The mechanism of action of those proteins may be autocrine, paracrine, or distant from AT [9]. To date, several AT types are identified, i.e., white (WAT), brown (BAT), and beige (BGAT) distributed in various anatomical parts throughout the organism [6]. Adult canines contain AT located mainly in subcutaneous and visceral depots [10]. In regenerative veterinary medicine (RVM), AT from the periovarian area, ligament falciform, and subcutaneous area is generally used. It can also be easily obtained during elective surgeries such as ovariotomy and gastropexy where AT is collected as medical waste (Figure 1).

## 2. Adipocytes—The Main Compound of AT

Adipocytes are the main compounds of AT and can exist in almost every organism structure. They occur individually or in small groups scattered throughout the connective tissue. Loose connective tissue contains adipocytes or clusters of multiple cells, but the tissue is referred to as AT when the fat cells outnumber other cell types [11].

WAT cells are formed soon after birth, and their main purpose is to store triglycerides. The formation of adipocytes starts with mesenchymal stem cells turning into adipoblasts which further differentiate into pre-adipocytes. After pre-adipocytes reach growth arrest, they change their appearance, accumulate triglycerides, and become mature adipocytes with lost ability of division [12]. BAT cells develop before birth and specialize in defending mammals against hypothermia [13]. The morphogenetic protein (BMP)-7 is responsible for the differentiation process of brown pre-adipocytes into BAT [14].

BAT is equipped with the metabolic machinery comprising the numerous mitochondria and the appropriate enzymes that allow fatty acids to oxidize at enhanced rates than that of WAT. In addition, the mitochondria of brown adipose tissue cells primarily generate heat rather than adenosine triphosphate (ATP) and can sustain body heat during prolonged periods of cold [15].

The last discovered and current highly researched type of adipocytes are beige orbrite. These have the characteristics of WAT and BAT cells [16]. The synthesis of BGAT is a highly investigated topic in diabetes and metabolism research [17,18].

As mentioned, AT was once viewed as a passive triglyceride depot, but AT is now known as a complex tissue giving residence to various interacting cells, also known as the stromal vascular fraction (SVF) [1,13].

## 3. Stromal Vascular Fraction—Interacting Cells Hosted by AT

**Figure 2:**
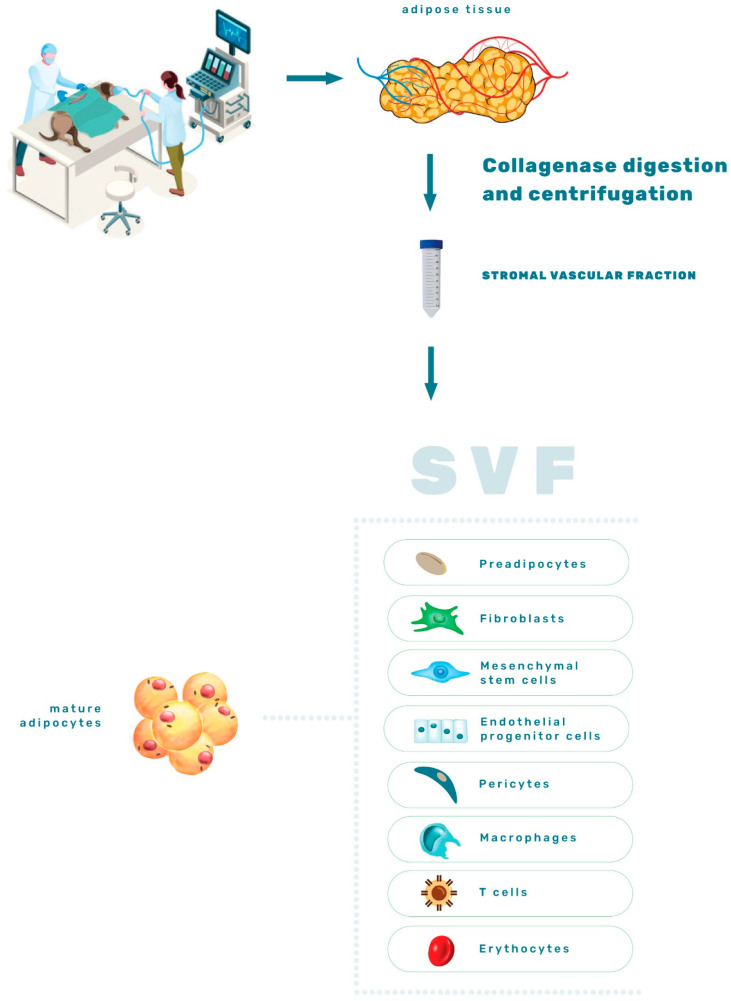
Graphical representation of the stromal vascular fraction components.

SVF is an eclectic combination of cells, including adipose-derived mesenchymal stem cells (ASCs), blood cells, endothelial precursors, endothelial and smooth muscle cells, pre-adipocytes, pericytes, macrophages and adipocytes [3,19,20] (Figure 2). Although adipocytes account for >90% of AT volume, SVF predominates in overall cell number [13]. In humans, SVF cells isolated from WAT possess more hematopoietic cells, macrophages, hematopoietic progenitors, and immature cells that, together, contribute to a higher degree of plasticity than SVF cells isolated from BAT [3,21].

**Figure 3:**
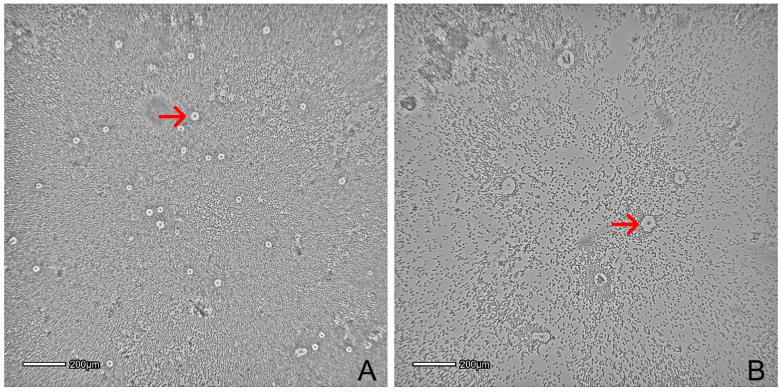
(**A**,**B**) represent stromal vascular fraction 24 h post isolation from peri-ovarian and subcutaneous adipose tissue, seeded in T25 flask after mechanical and enzymatic disruption. Cells pointed with a red arrow are plastic adherent cells in expansion; the surrounding cells are nonadherent. Pictures were obtained with Cytosmart Lux2 (CytoSMART Technologies B.V., The Netherlands).

Isolation of SVF from AT can be obtained by mechanical disruption and enzymatic digestion (Figure 3). The AT disassociation and SVF extraction most often involve a combination of the mechanical disruption of connective tissue, followed by enzymatic digestion with collagenase [22,23]. Both procedures aim to preserve the stem cells, the vascular compartment (stromal cell niche) viability and the therapeutic benefits of SVF products [24]. However, there are differences in outcomes between these two methods. For example, enzymatic digestion provides the phenotype of individual cells, while the mechanical extraction itself preserves interaction between cells and matrix [25]. Nevertheless, enzymatic digestion is considered the “gold standard” since it provides significantly greater cell viability [26].

The tissue harvesting site also presents a challenge since it can impair SVF and ASCs viability, cellular yield and immunophenotype. Recently, Hendawy et al. (2021) found that the peri-ovarian region is the most favourable site for harvesting ASCs in dogs since it contains the highest number of viable cells per gram of AT compared to subcutaneous and falciform ligament sites and also the highest number of CD90+ cells [22]. In 2013, Astor et al. reported similar results; AT collected at the falciform location had significantly fewer viable cells per gram (VCPG) than tissue collected at the thoracic wall and inguinal sites [27]. The same authors also reported the influence of age in SVF cell viability with significantly higher VCPG in dogs up to 4.5 years old; higher VCPG was also noted in non-spayed dogs compared to spayed ones. In addition, other authors noted the significantly higher population doubling and differentiation potentials in young donors [28]. As observed, consideration of many specific factors is needed to provide the best SVF therapy solution.

### 3.1. Mechanism of Action

The existing literature suggests that SVF achieves regeneration and healing through pro-angiogenic and immunomodulatory mechanisms, including differentiation and extracellular matrix secretion [19]. The first study reports the effectiveness of SVF therapy in dogs in 2007 [29]. The effectiveness may be due to the presence of ASCs, the vascular niche cells, and, finally, the interactions between all cells present in SVF [24]. Senesi et al. (2019) retain that the anti-inflammatory and immunoregulatory effect of SVF for osteoarthrosis is more likely than cells’ ability to differentiate in the specific cell lineage [26]. Hendawy et al. (2021) attribute the crucial effects of SVF to the presence of a sufficient number of ASCs, with preserved differentiation capacity. Because of the complex interactions between SVF and specific organs, the function of SVF in the treatment of various pathologies needs further clarification [22,26]. The mentioned mechanisms of action are elaborated in detail in the following sections of this review.

### 3.2. SVF Clinical Application for Various Conditions

Adipose SVF injection proved helpful in the orthopaedic field because it is a favourable, minimally invasive, non-surgical alternative for treating musculoskeletal disorders [26]. Osteoarthritis of the hip joint was significantly improved 24-weeks following treatment with simultaneous intraarticular (IA) and intravenous (IV) injection of autologous adipose-derived SVF and platelet-rich plasma (PRP) [30]. Lameness and range of motion significantly improved, as well as the overall quality of life in a double-blind study of canine hip joint osteoarthritis after 30, 60 and 90 days; although the cells in this study are named ASCs, the study indicates the application of a heterogeneous population of cells, including ASCs [29]. The same research group tested dogs suffering from elbow joint osteoarthritis. The placebo control group was not included in this study, but based on their previous analysis of hip joints, the significant improvement was attributed to the IA injected AT-derived heterogeneous cell population [31]. In four canine patients diagnosed with hip dysplasia, autologous SVF acupoint injection showed marked improvement, compared with baseline results after the first week of treatment [32].

The use of allogenic SVF in degenerative joint disease of the spine in dogs revealed an increased serum level of the vascular endothelial growth factor of affected animals in the second week of treatment. In the eighth week, the levels were decreased [33]. The same study published that decreased pain and reduced lameness were noticed a few days following therapy, overall concluding the improvement of joint regeneration capacity. The lack of research in the veterinary field indicates a significant need for further investigation of SVF benefits.

## 4. Adipose-Derived Mesenchymal Stem Cells—An Outstanding Component of the SVF

It is well known that stem cells provide tissues and organs with a fresh cellular compartment that can replace cells that have expired naturally and provide physiological balance in the organism. In addition, the expiration of cells due to natural processes or damage enables regeneration of the tissues [34]. The significant discovery of a stem cell system within AT occurred twenty years ago [35,36]. This finding raised considerable interest in the veterinary scientific community. The results were first documented in 2008 when scientists successfully isolated and fully described ASCs in canines [37] which laid the foundation for RVM.

**Figure 4:**
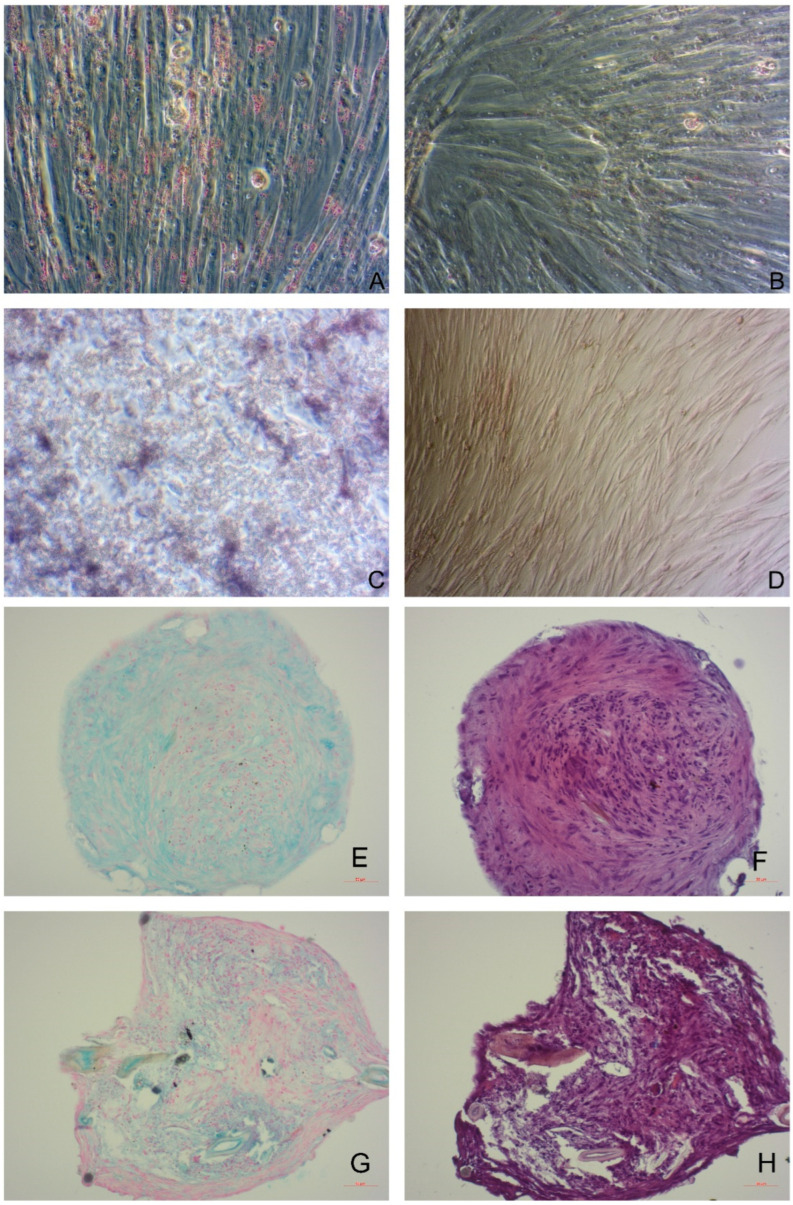
(**A**,**B**) Successful differentiation of canine adipose-derived mesenchymal stem cells (ASCs) in adipocyte differentiation media. When stained with Oil O Red, accumulated lipid droplets show high-intensity red staining within the cell (**A**) regarding control cultivated in basal medium (**B**). (**C**,**D**) Canine ASCs after successful osteodifferentiation; cells were stained with substrate to detect alkaline phosphatase activity. Purple strains of canine ASCs showed activity of alkaline phosphatase (**C**), while cells cultivated in basal medium (negative control) (**D**), showing low-intensity staining. 4E-H Images of histological sections of paraffin-embedded spheroids (20×, Zeiss, Germany) of canine ASCs three-dimensional culture after successful chondrodifferentiation. ASCs spheroids were stained with Alcian blue to detect the presence of aggrecan (**E**,**G**) and with H&E (**F**,**H**). Microscopic images (20×) (**A**–**H**) were taken with Zeiss Axiovert, Carl Zeiss AG, Jena, Germany.

The ASCs, a subpopulation within SVF, are non-hemopoietic stem cells originating from the mesoderm [38]. What makes them intriguing for cell research and therapy, among MSC properties such as self-renewal, in vitro proliferation, non-specialization, and ability to differentiate in another type of cell, is their easy accessibility. To address AT isolated cells as ASCs, the International Society for Cellular Therapy (ISCT) and The International Federation for Adipose Therapeutics (IFATS) have provided guidelines and recommendations for the minimal essential characterization of human ASCs. The established criteria were: capacity to proliferate as adherent cells in cultures, the ability of minimal three lineages in vitro differentiation (osteogenic, chondrogenic and adipogenic) (Figure 4), phenotypical positivity for CD90, CD73, CD105 and negativity for CD14, CD34, CD45, CD11b, CD19 or CD79α [24,39]. Although scientists apply those rules for canine ASCs research, the exact criteria are still not wholly established for this species. Though, numerous studies are contributing to ASCs characterization. In this context, the investigation of these changes in surface marker expression (CD73, CD90, CD29, CD44, CD271, CD45 and CD14) has been performed through six passages, providing a timeframe the ASCs cultivated in vitro possess optimal surface marker expression for use in therapy [23].

From the moment of their discovery, ASCs features were exploited in vitro to generate sufficient cell numbers to reach therapeutic doses depending on the disease for which the ASCs are being tested; meanwhile, their properties, gene expression and surface marker expression can be heavily influenced by such manipulation. Inevitably, prolonged cultivation in vitro carries side effects in terms of affection of the characteristic ASCs membrane markers responsible for their positive impact [23]. Therefore, basic research on ASCs properties in vitro is needed to further reveal their molecular signatures.

### 4.1. Mechanism of Action 

As already well documented for MSC in general, the healing properties are probably a result of the secretion of many factors influencing the immune system, with anti-apoptotic, anti-inflammatory, chemotactic and pro-angiogenic functions [10,40,41,42]. 

**Figure 5:**
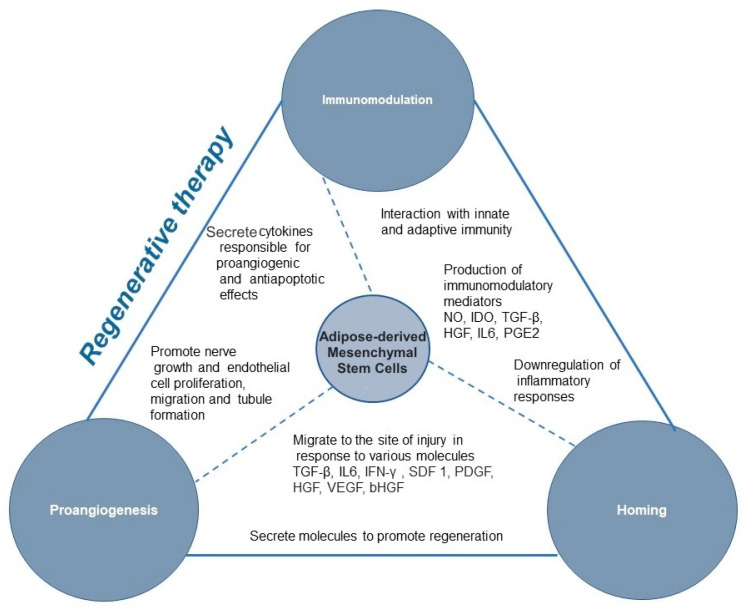
Schematic representation of adipose-derived mesenchymal stem cell features explored within regenerative therapy.

The mechanism of action (Figure 5) operates by sending and receiving autocrine, paracrine, endocrine and intracellular signals [40]. However, the primary therapeutic effect of MSCs is paracrine signalling inducing functional changes in monocytes/macrophages, dendritic cells, T-cells, B-cells, and natural killer cells [42]. Furthermore, MSCs can transfer various molecules through the extracellular vesicles (ECV): exosomes, microvesicles, and apoptotic bodies. ECVs are vesicles produced from the plasma membrane, and carry mRNA, proteins, miRNA, and mitochondria and travel within the body [42]. Except for the two MSC mechanisms of action mentioned above, apoptosis-mediated immunomodulation and mitochondrial transfer are other possible mechanisms of MSC action [42].

#### 4.1.1. Immunomodulation

The interaction of MSCs with the innate and adaptive immune systems usually results in the downregulation of ongoing inflammatory responses, though the immune response can also be upregulated. The MSC immunomodulation is influenced by many factors such as activation, tissue of origin, dose and time of application, and interaction with immune cells [43]. MSC immunomodulation remains yet to be elucidated; however, paracrine signalling via immunomodulatory mediators such as nitric oxide (NO), indoleamine 2,3-dioxygenase (IDO), transforming growth factor-β (TGF-β), hepatocyte growth factor (HGF), hemoxygenase (HO), IL-6 and prostaglandin E2 (PGE2) is believed to be the first stage. In addition, this may also occur through direct contact between cells [43,44,45]. Chow et al. (2017) reported that canine MSC suppressed T cell activation by TGF-b signalling pathways and adenosine signalling [46]. This finding further indicates that canine MSC, unlike human and rodent MSC, relies primarily on cyclooxygenase and TGF-b pathways for T cell suppression rather than on NO or IDO-mediated pathways. Besides suppressing T cells, MSCs suppress B cell activation and proliferation, dendritic cells maturation, inhibit NK cell proliferation and cytotoxicity, and promote regulatory T cell generation via soluble factors or cell-cell contact [44]. T cell necrosis by canine MSC is an additional mechanism of immune modulation [46]. Canine ASCs can suppress lipopolysaccharide mediated activation/maturation of canine dendritic cells (DC). The impact in vivo of such squelched DC activation would undoubtedly result in an attenuated ability to appropriately prime T cell responses. This effect would be exacerbated if the ASCs were first activated with IFNg, suggesting that the suppressive effect would be optimal in an inflammatory environment typical of autoimmune or pro-inflammatory conditions [47].

Another “immune-privileged” MSC property is their low immunogenicity attributed to low expression of MHC I, absence of co-stimulating CD80, CD86 and CD40, MHC II deficiency and whole paracrine spectrum of biomolecules and growth factors through which they establish their action [48,49,50]. Each of the mentioned pathways reflects the possibilities these cells offer to treat various disorders and organ systems. However, all aforementioned mechanisms also imply the differences between species and offer space for new acknowledgements.

#### 4.1.2. Homing

The MSCs have a remarkable ability to locate damaged tissues [3,42]. In response to chemotactic signals, MSCs reach the circulation and migrate to the site of injury, where they secrete molecules to promote regeneration. However, it is unclear which chemotactic signals guide MSCs to appropriate microenvironments [51]. The homing of MSCs is currently inefficient, and after they are systemically administered a small percentage of cells reach the target tissue [52]. The process of migration from the bloodstream to tissue involves steps for lymphocyte migration: (1) tethering and rolling, (2) activation, (3) firm adhesion, (4) transmigration or diapedesis, resulting in migration into tissue due to chemotaxis as described by Sackstein [53]. The migration of MSCs occur in response to various chemokines and growth factors, including TNF-α (tumour necrosis factor-α), IL-6, IL-8 [54]. Unlike comprehensive knowledge on blood cell homing, MSC homing remains poorly understood as tethering or rolling and transmigration.

The therapy research of MSCs bears one of the most significant aims, i.e., improving their homing efficiency. MSC homing can be categorized into (1) targeted administration—administration of ASCs at or near the target tissue, (2) magnetic guidance—cells labelled with magnetic particles are directed to the organ of interest using an external magnetic field, (3) genetic modification—permanent overexpression of homing factors via viral transduction, (4) cell surface engineering—temporarily chemical engineering by enzymes or ligands, (5) in vitro priming—altering culture conditions to affect gene expression, (6) and modification of the target tissue by direct injection of homing factors, genetic modification of target tissue, scaffold implantation, or using radiotherapeutic and ultrasound techniques[52].

#### 4.1.3. Pro-Angiogenic and Anti-Apoptotic Mechanism of Action

MSCs secrete various cytokines responsible for pro-angiogenic and anti-apoptotic effects and in doing so, MSCs enable tissue regeneration and revascularization. Soluble angiogenic factors secreted by MSCs include fibroblast growth factors, hepatocyte growth factor and the vascular endothelial growth factor.

The lack of canine-specific antibodies has hampered identification of growth factors in the secretome of canine MSCs. Likely, secretomes of other species are similar to secretomes secreted by canine MSCs thus there is an idea on the secretome composition of canine MSCs based on this information [55]. Canine MSCs promote nerve growth and endothelial cell proliferation, migration and tubule formation by secretion of neurotrophic and angiogenic factors. Delfi et al., 2016 demonstrated MSC paracrine activity on nerves and blood vessels in the vicinity of the wound site. It was shown that MSC transplants promote increased neuronal function in dogs with central nervous system damage [55]. The following study by the same authors, revealed that the conditioned medium from human and canine MSCs cultures exhibited neurogenic and angiogenic effects and increased SH-SY5Y neuronal proliferation, βIII tubulin immunoreactivity, neurite outgrowth, and EA.hy926 endothelial cell proliferation, migration and the formation of endothelial tubule-like structures, to a significantly greater extent than control medium, indicating marked trophic activity [55].

Regarding anti-apoptotic action, it was shown that ASCs protect against radiation-induced dermatitis by exerting an anti-apoptotic effect through inhibition of cathepsin F (CTSF) expression. In addition, ASCs markedly attenuated radiation-induced apoptosis, downregulated CTSF and downstream pro-apoptotic proteins (Bid, BAX, and caspase 9), and upregulated anti-apoptotic proteins (Bcl-2 and Bcl-XL) [56].

### 4.2. ASCs Clinical Application for Various Conditions

**Figure 6:**
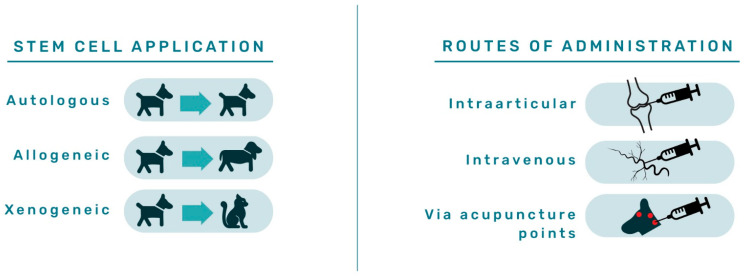
Graphical presentation of canine adipose-derived mesenchymal stem cell application strategies and routes of administration applied within available studies described in the literature.

Since their discovery, the outstanding properties of ASCs have been continuously tested in numerous diseases in dogs. The application of stem cells for therapy can be autologous; when a patient receives their cells, allogeneic therapy refers to cells derived from a donor of the same species as the receiving animal and xenogeneic therapy refers to application of donor cells of a different species. The routes of administration (Figure 6) are most often diverse, but IA, IV, and administration via acupuncture points are most frequently used, as extensively reviewed by Brondeel et al. in 2021 [57].

**Figure 7:**
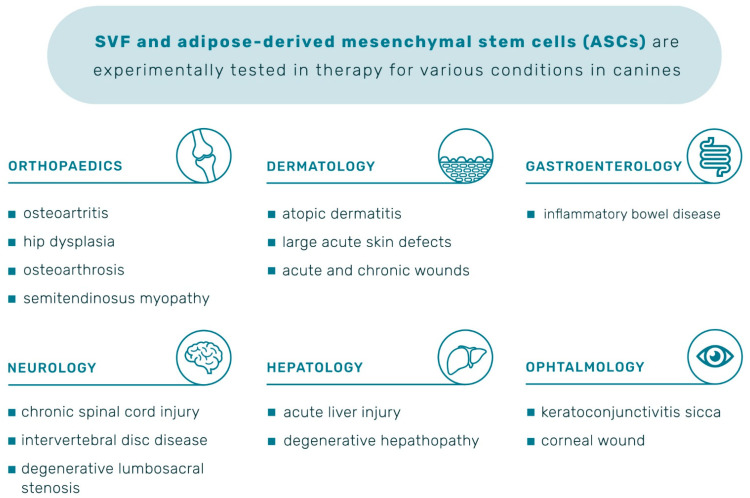
Pathological conditions in canines for which adipose-derived mesenchymal stem cell therapy was applied.

Clinical trials in which mesenchymal stem cells are used on dogs are available on the first registry set up for animal studies, preclinicaltrials.eu, launched in April 2018. The chronically systematic order of positive outcomes of ASCs therapy for various conditions is presented in Table 1 and a graphic summary in Figure 7. Detail description of the effects within those studies is described in the following sections. 

#### 4.2.1. Orthopaedics

Therapeutic effects of autologous and allogeneic ASCs applications in orthopaedics have improved pain and lameness in dogs with osteoarthritis [57,58,60,61,64,65,67,68,69,70]. In 2018, a group of scientists described the allogeneic ASCs application on 203 dogs and concluded that IA treatment gave better results when compared with the IV treatment in the polyarthritis condition. The age proved influential as most dogs under the age of five receiving IA treatment showed good improvement [67]. In addition, the canines’ overall health and vitality are significant factors in response to the ASCs therapy. Positive therapeutic outcomes were further observed in chronic osteoarthritis (OA) of the hip and elbow joint [59,63,66]. In addition, ASCs treatment significantly improved the symptoms of hip dysplasia in 60% of treated dogs after one week [32]. This study compared the effects of SVF and ASCs therapy administered to acupuncture points and reported better results for SVF than ASCs therapy. However, it was concluded that SVF or allogeneic ASCs could be safely used as an acupoint injection for treating hip dysplasia in dogs [31]. A follow-up study highlighted the importance of cell administration before the injury becomes severe [59]. Furthermore, significant improvements following ASCs therapy of semitendinosus myopathy are documented [71,72].

#### 4.2.2. Neurology

In dogs with chronic spinal cord injury/intervertebral disc disease, percutaneous intraspinal transplantation of allogeneic ASCs had no adverse effects or complications (infection, neuropathic pain, or worsening neurological function) during the 16-week follow-up period. In addition, three animals improved locomotion, and one animal walked without support. However, no changes in deep pain perception were observed [73]. In the most recent research on lumbosacral spinal cord injury, transplantation of allogeneic ASCs with surgery in four dogs showed significant neurological improvements with normal ambulatory ability (4/4) and urinary control (3/4) three months after the surgery and the first ASCs transplantation [76]. While in the case of acute paraplegia, epidural canine ASCs transplantation with surgical decompression contributed to faster locomotor recovery and reduced the length of post-surgery hospitalization [74]. Another successful case reported the use of cultured autologous ASCs injected bilaterally at the level of L7-S1 in the external aperture of the intervertebral foramen of degenerative lumbosacral stenosis in a canine patient [75].

#### 4.2.3. Dermatology

The stem cell treatment also gained popularity in treating skin pathologies; systemic administration of ASCs had a positive outcome for atopic dermatitis refractory to conventional medications for six months and with no side effects [78]. The prospective role in dermatology was also shown in treating large acute skin defects when corrective surgery offers no solution, as Zubin et al. (2015) [77] reported. In addition, the healing of acute and chronic wounds in 24 dogs of different ages and breeds significantly improved in a manner of contraction and re-epithelialization in the treated group. Furthermore, histopathological findings revealed an inflammatory infiltrate decrease and the presence of multiple hair follicles on day seven after treatment with ASCs [79]. Most recently, Kaur et al. (2022) performed the first double-blinded, placebo-controlled evaluation of the efficacy of allogeneic canine ASCs to treat canine atopic dermatitis. No severe side effects were observed in any patient in this study. Furthermore, the high dose ASCs treatment proved to be efficacious in alleviating the clinical signs of atopic dermatitis until 30 days after the last subcutaneous administration of MSCs[80].

#### 4.2.4. Ophthalmology

Reviewing ophthalmological benefits, an immune-mediated condition common in humans and canines, keratoconjunctivitis sicca (KCS), was studied. Results in canines with KCS revealed that a single infusion of ACSs into lacrimal glands of 15 dogs resulted in no side effects during 12-months follow up.

Furthermore, a significant clinical improvement was observed in all patients, single administration was effective, and daily use of corticosteroids was not required [82]. In 2019, topical application into the conjunctival sac resulted in decreased expression of pro-inflammatory markers, which implies ASCs as an adjuvant therapy in treating KCS in dogs and humans. [83]. Another successful study of ASCs for KCS was reported with significant outcomes in canines where allogeneic ASCs were applied [81]. Falcao et al., in 2020, evaluated the use of sub-conjunctival applied ASCs in dogs diagnosed with deep corneal ulcers. Allogeneic ASCs therapy in 22 out of 26 dogs presented complete ulcer wound healing within 14 days, totalling 84.6%, indicating that this therapy is a simple solution to substitute surgery with satisfying results [84].

#### 4.2.5. Gastroenterology

The ASCs therapy was also tested for currently uncurable inflammatory bowel disease (IBD); administration of a single IV ASCs infusion showed no acute reaction or side effects during the follow-up of 11 dogs. Furthermore, 9 out of 11 dogs were in clinical remission. As the primary goal of treatment is to reduce symptoms, achieve and maintain remission, and prevent complications, ASCs were well tolerated and appeared to produce clinical benefits in dogs with severe IBD [85].

#### 4.2.6. Hepatology

Liver diseases share clinical and pathological features in humans and canines, thus, dogs may be a representative model for humans. Autologous ASCs transplantation in dogs with liver diseases significantly ameliorated liver function; decreased liver biomarkers and observed effects seem to be related to stem cells’ immunomodulatory mechanism of action [87,88]. The effects of allogenic ASCs on acute liver injury by carbon tetrachloride in dogs were investigated by Teshima et al. (2017). It was observed that serum liver enzymes decreased significantly. In the liver, the mRNA expression levels of pro-inflammatory cytokines, such as IL-1, IL-6, IL-8, and IFNγ decreased significantly, but anti-inflammatory cytokines such as IL-4 and IL-10, HGF and VEGFA, were significantly increased after the first ASCs injection. The authors suggest that allogenic ASCs ameliorate acute hepatic injury in dogs [86].

## 5. The Importance of the Regulatory Considerations and Safety Aspects Using of Animal Cell-Based Products in Regenerative Therapy

As demonstrated by these positive examples, the use of ASCs therapy for numerous conditions holds excellent promise and encourages more research to provide safe, effective, and quality treatment. The European Medicines Agency (EMA), 2015, published the first draft problem statement agreed by Ad Hoc Expert Group on Veterinary Novel Therapies (ADVENT), which raised questions concerning the sterility of animal-cell-based products. The conclusion, published in 2019, was that sterility assurance of the finished stem-cell product is critical in light of the fact that the product may be administered prior to final sterility result being obtained [89]. The Novel Therapies and Technologies Working Party (NTWP) of the EMA Committee for Medicinal Products for Veterinary Use is currently preparing scientific guidance on the requirements for authorization of novel therapy veterinary medicines, which involves guidelines on veterinary cell-based therapy products taking into consideration the mechanism of action, potency and clinical effects [90]. In order to implement safe new treatments for animals, the FDA published guidelines for the application and handling of animal-cell-based products, and all cell-based products require premarket review and FDA approval to be legally marketed. Precautionary steps in therapeutic use include the control of transmitting infectious agents, tumorigenicity or unintended tissue formation, immunogenicity, long-term safety, cell survival and biodistribution [91].

## 6. Conclusions

To conclude, while AT was once considered an energy depot, today it is well known that, among others, AT hosts components with extraordinary potential in relieving pain and treating numerous diseases. The canine SVF and ASCs treatments provide many benefits, starting with the degenerative orthopaedic pathologies, and also regenerative possibilities within other organs such as skin, bowel, and eyes. In addition, although this review focused on positive aspects of therapy in canines, the possible side effects it can carry should not be overlooked, such as the transmission of infectious agents, tumorigenicity, immunogenicity, donor selection, long-term safety, cell survival, biodistribution or ectopic tissue formation [91]. The (NTWP) of the EMA Committee for Medicinal Products for Veterinary Use is currently preparing scientific guidance on the requirements for authorization of novel therapy veterinary medicines, which involves guidelines on veterinary cell-based therapy products taking into consideration the mechanism of action, potency and clinical effects [90].

Although therapy will be available in the near future, there remains much laboratory and clinical work to undertake to better understand the complexity behind the healing mechanisms of canine ASCs. In this context, the development of regenerative veterinary medicine is essential not only for pets and their health but also for humans since canines represent an important model for human conditions. Nevertheless, it is evident that the course of research in this field is expanding, which welcomes further high quality basic, translational, and clinical research in stem cell regenerative therapy. Furthermore, in order to positively impact the lives of canine patients, adequate research for safe and standardized treatment is a fundamental prerequisite.

## Figures and Tables

**Table 1:** Studies of canine adipose-derived mesenchymal stem cells (ASCs) and stromal vascular fraction (SVF) applied in canine pathological conditions.

	Clinical Condition	Number of Canines Included	Type of Application	Route of Administration	Number of Cells (×10^6^)	Reference
**Orthopaedics**	Osteoarthritis of hip joints	21	Autologous SVF	Intraarticular	4.2–5	Black et al. (2007) [29]
	Osteoarthritis of the elbow joint	14	Autologous SVF	Intraarticular	3–5	Black et al. (2008) [31]
	Stifle joint osteoarthrosis	1	Autologous ASCs + hyaluronic acid	intraarticular	1	Yoon et al. (2012) [58]
	Chronic osteoarthritis of the elbow joints	4	Autologous ASCs + hyaluronic acid/PRP	Intraarticular	3–5	Guercio et al. (2012) [59]
	Osteoarthritis of hip joints	13	Autologous ASCs + PRP	Intraarticular	15	Vilar et al. (2013) [60]
	Osteoarthritis of hip joints	18	Autologous ASCs	Intraarticular	30	Cuervo et al. (2014) [61]
	Hip dysplasia	SVF = 4 ASCs = 5	Autologous SVF or allogeneic ASCs	Acupoint injection	SVF = 2–5 ASCs = 0.2–0.8	Marx et al. (2014) [49]
	Osteoarthritis of hip joints	15	Autologous ASCs	Intraarticular	15	Vilar et al. (2014) [62]
	Osteoarthritis of hip joints	22	Autologous SVF + PRP	Intraarticular and intravenous	N/A	Upchurch et al. (2016) [30]
	Osteoarthritis of different joints	74	Allogeneic ASCs	Intraarticular	12	Harman et al. (2016) [63]
	Surgical-induced osteoarthritis in Beagle dogs	24	ASCs and/or PRP	Intraarticular	10	Yun et al. (2016) [64]
	Osteoarthritis of hip joints	15	Autologous ASCs	Intraarticular	15	Vilar et al. (2016) [65]
	Osteoarthritis of the elbow joint	30 (39 elbows)	Allogeneic ASCs + hyaluronic acid	Intraarticular	12 ± 3.2	Kriston-Pal et al. (2017) [66]
	Osteoarthritis and other joint defects	203	Allogeneic ASCs	Intraarticular and/or intravenous	N/A	Shah et al. (2018) [67]
	Osteoarthritis of different joints	10	Autologous ASCs	Intraarticular	15–30	Srzentić Dražilov et al. (2018) [68]
	Osteoarthritis of the elbow joint	13	Allogeneic ASCs	Intravenous	1–2/kg body weight	Olsen et al. (2019) [69]
	Osteoarthritis of hip joints	12 (24 hips)	Allogeneic ASCs	Intraarticular	5	Wits et al. (2020) [70]
	Acute semitendinosus muscle injury	2	Autologous SVF	Intramuscular and intravenous	4.7	Brown et al. (2012) [71]
	Semitendinosus myopathy	11	Autologous ASCs	Intramuscular and intravenous	N/A	Gibson et al. (2017) [72]
**Neurology**	Chronic spinal cord injury	6	Allogeneic ASCs	Intraspinal	N/A	Escalhao et al. (2017) [73]
	Acute thoracolumbar disc disease and spinal cord injury	22	Allogeneic ASCs	Epidural	10	Bach et al. (2019) [74]
	Degenerative lumbosacral stenosis	1	Autologous ASCs	Paravertebral and intraarticular	Paravertebral = 30.6 Intraarticular = 15.3	Mrkovački et al. (2021) [75]
	Lumbosacral spinal cord injury	4	Allogeneic ASCs + surgery	Nerve roots next to injury, intravenous and epidural	Nerve roots next to injury = 5 Intravenous = 4 Epidural = N/A	Chen et al. (2022) [76]
**Dermatology**	Large skin wound	1	Autologous ASCs + PRP	Local dripping or spraying	N/A	Zubin et al. (2015) [77]
	Atopic dermatitis	26	Allogeneic ASCs	Intravenous	1.5	Villatoro et al. (2018) [78]
	Acute and chronic skin wound	24	Allogeneic ASCs	Intradermal	30	Enciso et al. (2020) [79]
	Atopic dermatitis	15	Allogeneic ASCs	Subcutaneous	Low dose = 0.5/kg body weight High dose = 5/kg body weight	Kaur et al. (2022) [80]
**Ophthalmology**	Keratoconjunctivitis sicca	12	Allogeneic ASCs	Around the lacrimal glands	5	Villatoro et al. (2015) [81]
	Keratoconjunctivitis sicca	15 (24 eyes)	Allogeneic ASCs	Intralacrimal	1	Bittencourt et al. (2016) [82]
	Keratoconjunctivitis sicca	22	Allogeneic ASCs	Topic in the conjunctival sac	1	Sgrignoli et al. (2019) [83]
	Corneal wound	26	Allogeneic ASCs	Sub-conjunctival	3	Falcao et al. (2020) [84]
**Gastroenterology**	Inflammatory bowel disease	11	Allogeneic ASCs	Intravenous	2/kg body weight	Perez-Merino et al. (2015) [85]
**Hepatology**	Acute liver injury	9	Allogeneic ASCs	Peripheral vein/splenic vein	2	Teshima et al. (2017) [86]
	Degenerative hepatopathy	10	Autologous ASCs	Portal vein	0.5/kg body weight	Gardin et al. (2018) [87]
	Acute liver injury	6	Allogeneic ASCs	Intravenous	10	Yan et al. (2019) [88]

## Data Availability

Not applicable.
